# Complications and outcomes of hospitalizations for patients with and without Parkinson disease

**DOI:** 10.3389/fnagi.2023.1276731

**Published:** 2023-12-15

**Authors:** Benjamin P. George, William A. Barbosa, Anish Sethi, Irene H. Richard

**Affiliations:** ^1^Department of Neurology, University of Rochester Medical Center, Rochester, NY, United States; ^2^Drexel University College of Medicine, Philadelphia, PA, United States

**Keywords:** Parkinson disease, hospitalization, complications, outcomes, epidemiology, delirium, aspiration, mortality

## Abstract

**Objective:**

To examine complications and outcomes of hospitalizations for common indications for hospitalization among patients with Parkinson disease (PD).

**Methods:**

We identified and selected the ten most common indications for hospitalization among individuals ≥65 years of age using principal diagnoses from the California State Inpatient Database, 2018–2020. Patients with comorbid PD were identified using secondary diagnosis codes and matched one-to-one to patients without PD based on principal diagnosis (exact matching), age, gender, race and ethnicity, and Elixhauser comorbidity index (coarsened exact matching). We identified potentially preventable complications based on the absence of present on admission indicators among secondary diagnoses. In the matched cohort, we compared inpatient complications, early Do-Not-Resuscitate (DNR) orders (placed within 24 h of admission), use of life-sustaining therapies, new nursing facility requirement on discharge, and death or hospice discharge for patients with and without PD.

**Results:**

We identified 35,457 patients with PD among the ten leading indications for hospitalization in older adults who were matched one-to-one to patients without PD (*n* = 70,914 in total). Comorbid PD was associated with an increased odds of developing aspiration pneumonia (OR 1.17 95% CI 1.02–1.35) and delirium (OR 1.11 95% CI 1.02–1.22) during admission. Patients with PD had greater odds of early DNR orders [placed within 24 h of admission] (OR 1.34 95% CI 1.29–1.39). While there was no difference in the odds of mechanical ventilation (OR 1.04 95% CI 0.98–1.11), patients with PD demonstrated greater odds of tracheostomy (OR 1.41 95% CI 1.12–1.77) and gastrostomy placement (OR 2.00 95% CI 1.82–2.20). PD was associated with greater odds of new nursing facility requirement upon discharge (OR 1.58 95% CI 1.53–1.64). Patients with PD were more likely to die as a result of their hospitalization (OR 1.11 95% CI 1.06–1.16).

**Conclusion:**

Patients with PD are at greater risk of developing aspiration pneumonia and delirium as a complication of their hospitalization. While patients with PD more often have early DNR orders, they have greater utilization of life-sustaining therapies and experience worse outcomes of their hospitalization including new nursing facility requirement upon discharge and greater mortality.

## Introduction

Parkinson disease (PD) is a progressive, neurodegenerative disease that affects almost one million individuals in the United States, and approximately 8.5 million worldwide (Marras et al., 2018; Ou et al., 2021). Diagnosis of PD requires the presence of bradykinesia accompanied by either rest tremor, rigidity, or both (Tolosa et al., 2006; Bloem et al., 2021). With a projected increase in PD prevalence in the coming decades (Dorsey et al., 2013), it is important to understand the hospitalization burden of the disease and subsequent outcomes of inpatient stays (Yang et al., 2020). One-third of patients with PD experience hospitalization each year (Hassan et al., 2013). Direct medical costs of PD were estimated at $25.4 billion in 2017, which includes post-acute or long-term care, inpatient and outpatient care, medical equipment, prescriptions, and provider office visits (Yang et al., 2020). Inpatient costs for patients with PD comprised over one-quarter of all direct medical care costs at over $7.1 billion (Yang et al., 2020). Factors contributing to greater hospitalization rates in PD include gait abnormalities and orthostasis resulting in falls and injuries, autonomic dysfunction with altered urodynamics potentially leading to greater incidence of urinary tract infections, and dysphagia which can result in aspiration pneumonia (Aminoff et al., 2011; Gerlach et al., 2011; Bhattacharya et al., 2012; Martinez-Ramirez et al., 2015; Suttrup and Warnecke, 2016; Beydoun et al., 2017; Hogg et al., 2022).

In addition to factors that lead to hospitalization in patients with PD, specific health conditions and issues may arise after hospital admission that develop independent of the underlying illness or principal diagnosis (3M™ Health Care Academy, 2023). These complications may lead to unfavorable outcomes of hospitalizations for patients with PD (Gerlach et al., 2012). Venous thrombosis, bladder infections, delirium, and inpatient falls are all issues that may commonly be encountered in hospitalized patients with PD (Aminoff et al., 2011). The use of life-sustaining procedures, Do-Not-Resuscitate (DNR) orders, and discharge disposition in hospitalized patients may also differ for patients with PD (Mahajan et al., 2017). Few studies have used large population-based data to understand reasons for hospitalization, complications, and outcomes for patients with PD (Woodford and Walker, 2005; Mahajan et al., 2016). Additionally, to date, no studies have included hospice discharge when determining the risk of mortality for hospitalized PD patients, and though the Parkinson’s Foundation has recently identified dysphagia screening and aspiration pneumonia prevention strategies as recommended care standards for hospitalized PD patients (Parkinson’s Foundation Hospital Care Recommendations, 2023), a limited number of studies have investigated aspiration pneumonia as an inpatient complication for hospitalized patients with PD. Due to the potentially fatal risk of complications in hospitalized patients, it is important to elucidate which factors are associated with hospitalization for patients with PD.

We aimed to examine complications and outcomes of hospitalizations for common inpatient conditions among patients with PD compared to matched controls. We hypothesized that patients with PD are at an increased risk of complications and experience worse outcomes of hospitalizations.

## Methods

We used the California State Inpatient Database (SID) from the Agency for Healthcare Research and Quality, Healthcare Cost and Utilization Project (HCUP) (Overview of the State Inpatient Databases (SID), 2023) from January 1, 2018 to December 31, 2020 to perform a retrospective observational analysis of deidentified older adult patients admitted for any one of the ten most common reasons for hospitalization among individuals ≥65 years of age and compared complications and outcomes for those with and without Parkinson disease (PD). The University of Rochester Medical Center Research Subjects Review Board approved the study. The study adheres to the STROBE guidelines for the reporting of observational studies.

### Data source

There are 58 counties in the state of California, over 300 acute care hospitals throughout the state, with more than 3 million hospitalizations per year (State Inpatient Databases (SID) File Composition, 2023). The California SID includes a complete enumeration of all-payer administrative claims data on hospital discharges from all non-federal acute care hospitals within the state of California in each year. The SID includes patient demographics (age, sex, race and ethnicity, urban vs. rural origin, median household income for ZIP), primary and secondary diagnoses, procedures, and procedure timing (i.e., days from admission), as well as the length of stay, and detailed disposition including death. Race and ethnicity in the California SID are directly reported by HCUP partner organizations and consolidated by HCUP to uniform values which combine race and ethnicity into a single variable. In HCUP methodology, ethnicity took precedence over race. For example, if a patient was identified as Black and Hispanic, they were assigned to Hispanic. Additionally, HCUP consolidates some race categories (i.e., Asian and Native Hawaiian or Pacific Islander).

There is a single principal diagnosis for each hospitalization and up to 36 additional diagnostic fields representing chronic conditions and complications. There are 25 procedural fields each with a respective day of procedure indexed from day of admission. A small percentage of hospitalizations had a documented procedure code for all 25 procedural fields (0.05%). Diagnoses and procedures were identified using the International Classification of Diseases, 10th Revision, Clinical Modification (ICD-10-CM) and Procedure Coding System (ICD-10-PCS), respectively. We used secondary ICD-10-CM diagnoses to calculate an Elixhauser comorbidity index (Elixhauser et al., 1998).

The California SID is the only existing population-level database in the US that contains detailed administrative claims information and captures patient-level “Do-Not-Resuscitate” (DNR) status (Goldman et al., 2013). The presence of DNR status within the dataset indicates that a DNR order was written at the time of hospital admission as indicated by the California source documentation.

### Patient selection

We used Clinical Classification Software Refined (CCSR) from HCUP to aggregate ICD-10-CM codes into clinically meaningful categories (Clinical Classifications Software Refined (CCSR), 2023). We identified the 10 most frequent principal diagnoses by CCSR code for inpatient stays among hospitalizations ≥65 years of age: (1) Sepsis [CCSR INF002], (2) Heart Failure [CCSR CIR019], (3) Cerebral Infarction [CCSR CIR020], (4) Myocardial Infarction [CCSR CIR009], (5) Pneumonia [CCSR RSP002], (6) Acute Renal Failure [CCSR GEN002], (7) Cardiac Dysrhythmia [CCSR CIR017], (8) Hip Fracture [CCSR INJ006], (9) Urinary Tract Infection [CCSR GEN004], and (10) Chronic Obstructive Pulmonary Disease (COPD) [CCSR RSP008].

### Inclusion and exclusion criteria

**Figure 1:**
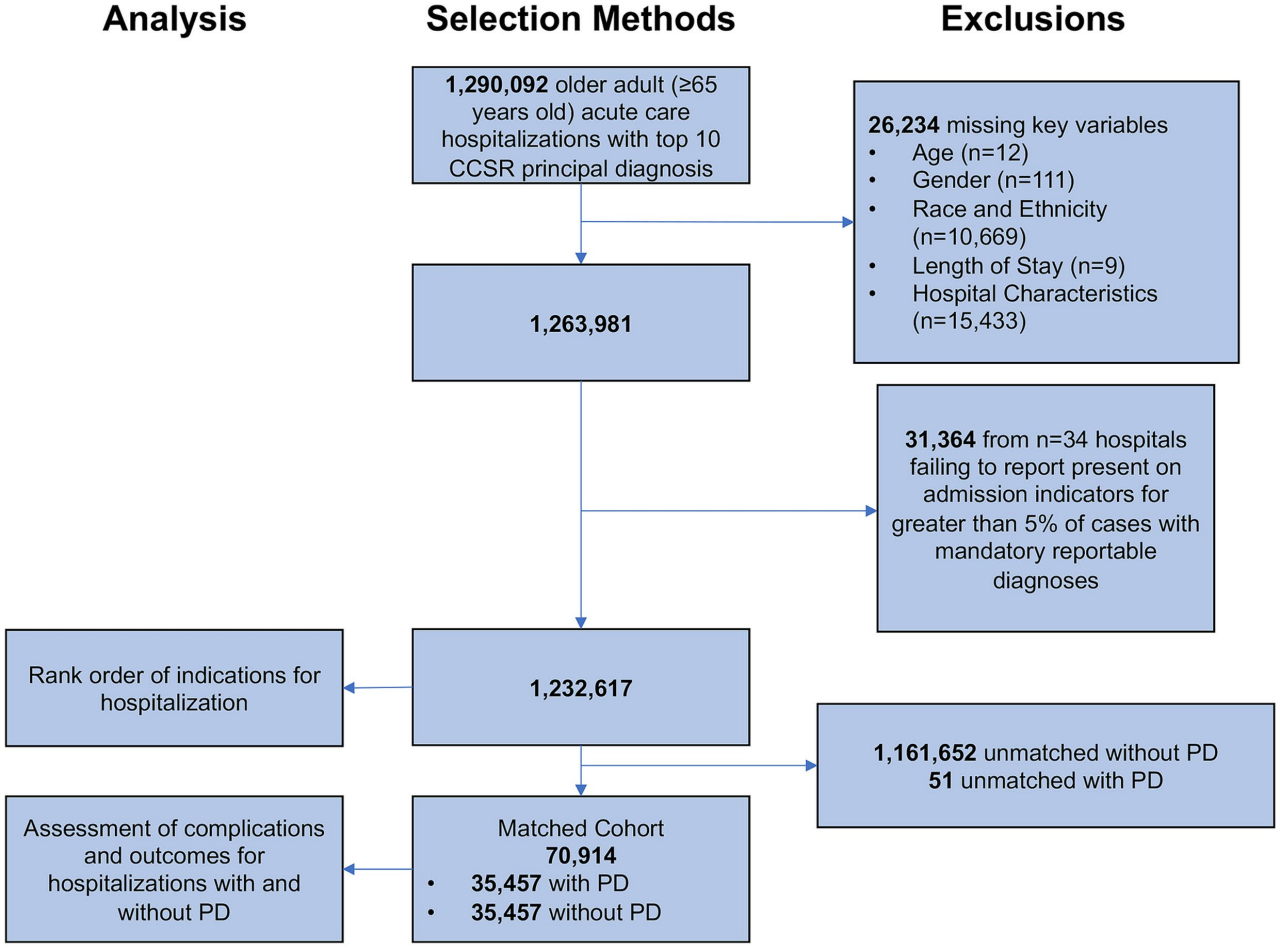
Selection methods. CCSR, Clinical Classification Software Refined. Patients with Parkinson disease were matched one-to-one to patients without Parkinson disease on principal diagnosis (exact matching), age, gender, race and ethnicity, and Elixhauser comorbidity index (coarsened exact matching).

The study included individuals ≥65 years of age admitted under non-elective, emergent circumstances to an acute care hospital in California between 2018 and 2020 for any one of the top ten principal diagnoses for older adults. Individuals <65 years of age were excluded from the analysis. Outgoing patient transfers from one acute care hospital to another acute care hospital were excluded from the analysis to avoid double counting the same patient. Observations with missing data for age, gender, race and ethnicity, length of stay, and hospital characteristics were excluded (Figure 1). Our study relies on the reporting of “Present on Admission” (POA) indicators to identify hospital complications. To refine our population and improve accurate identification of complications, our study excludes hospitals failing to report POA indicators for >5% of cases with mandatory reportable POA diagnoses (Hospital-Acquired Conditions (Present on Admission Indicator): Reporting, 2023).

### Parkinson disease

We used the ICD-10-CM code G20 present in any one of the 36 available secondary diagnoses to identify individuals with comorbid Parkinson disease.

### Potentially preventable complications

Potentially Preventable Complications (PPCs) are harmful events or negative outcomes (e.g., hospital-acquired aspiration pneumonia, deep venous thrombosis) that develop after hospital admission (while inpatient) and may result from care and treatment processes rather than the natural progression of the underlying illness and are, therefore, potentially preventable (Hughes et al., 2006; 3M™ Health Care Academy, 2023). We used ICD-10-CM/PCS codes to identify select PPCs among secondary diagnosis codes. Secondary diagnoses were considered PPCs if they met the coding criteria and did not have a “Present On Admission” (POA) indicator. The POA indicator is a data element on the Uniform Billing form for hospitalizations, available in the California SID, indicating if a diagnosis was present at the time of admission. The database contains diagnosis POA indicators which permit the identification of conditions that develop during the hospital stay (as opposed to those present on hospital admission). The presence of an “N” for POA indicator, specifying a hospital diagnosis was not present on admission, was used to positively identify complications of the hospitalization. We also made clinical exclusions of PPCs if complications were thought to be a natural consequence of the reason for hospitalization (e.g., aspiration pneumonia cannot be considered a complication for a hospitalization with a principal diagnosis of pneumonia), or if the complication were unlikely to occur within a short stay. These methods are similar to those previously used to define PPCs (Lagoe and Bick, 2013; 3M™ Health Care Academy, 2023).

### Outcome measures

We examined the number of select PPCs: aspiration pneumonia, *C. difficile* infection, deep venous thrombosis (DVT), pulmonary embolism, decubitus ulcer, delirium, ileus and other reduced motility gastrointestinal complications, inpatient fall, urinary tract infection, and in-hospital cardiac arrest. These PPCs were selected by the authors based on their relevance for patients with Parkinson disease considering potential contributions from dysphagia, immobility, encephalopathy, constipation, gait dysfunction, and frailty. PPCs and the respective ICD-10-CM/PCS codes used for the purposes of our study, as well as any relevant exclusions, can be found in Supplementary Table S1. We also examined utilization of life sustaining therapies (LSTs) including invasive mechanical ventilation, tracheostomy, and gastrostomy placement. LSTs and their respective ICD-10-PCS codes used in this study can be found in Supplementary Table S2.

We assessed hospital length of stay as reported by HCUP. New nursing facility requirement on discharge was defined as the presence of skilled nursing facility discharge based on the discharge data element without an indicator of nursing facility point-of-origin on the UB-04 claim form (Official UB-04 Data File, 2023). Death was defined as inpatient mortality or discharge to hospice (i.e., total mortality equivalence) (Asch et al., 2021).

### Statistical analysis

We compared the rank order of indications for hospitalization among patients with and without PD in the unmatched cohort using a chi-squared test.

Coarsened Exact Matching (CEM) was used to match patients with and without PD in a one-to-one ratio, to make the outcomes in both groups more comparable. CEM involves temporarily coarsening continuous data into predefined set-width bins, matching of categorical and binned-continuous variables of interest, and then running analyses on the uncoarsened matched data following the matching procedures (Blackwell et al., 2009). Patients were matched on age, gender, race and ethnicity, and Elixhauser comorbidity index. Matching was stratified by principal diagnosis to ensure comparisons were exact across indications for hospitalization.

Categorical variables were evaluated using chi-squared test. Continuous variables were found to be non-normal in distribution, and therefore, a Wilcoxon Rank-Sum test was used for comparisons. Conditional logistic regression was performed to calculate the odds associated with PPCs, LSTs, death, and new nursing facility requirement for patients with PD among matched subjects, reported as odds ratios compared to those without PD. Mortality was examined across subgroups by indication for hospitalization. Statistical significance was set *a priori* at *p* < 0.05. Analyses were performed using Stata version 18.0 (College Station, TX).

## Results

### Indications for hospitalization

**Figure 2:**
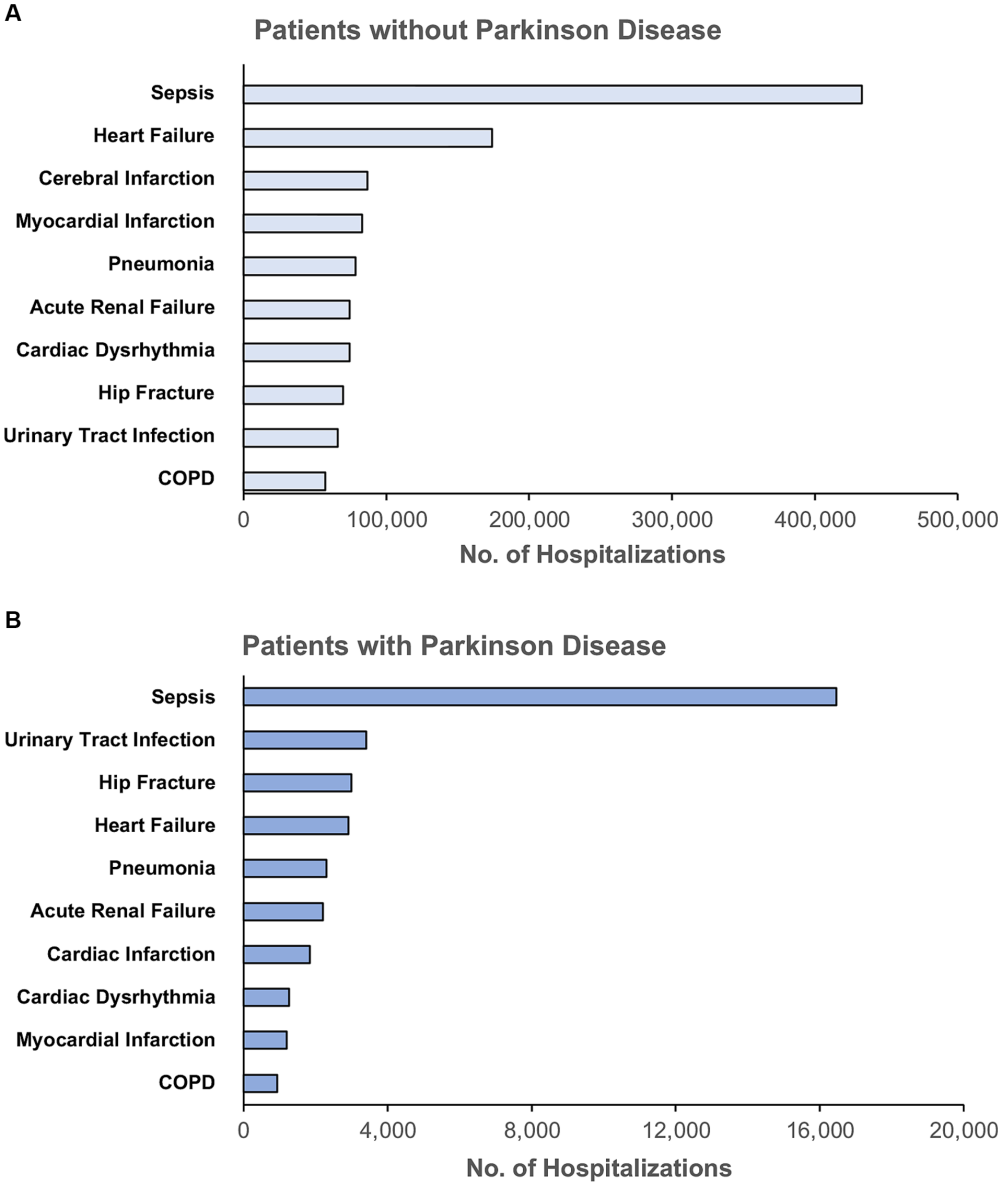
Rank order of the top ten indications for hospitalization among unmatched older adults **(A)** without Parkinson disease and **(B)** with Parkinson disease, unmatched. COPD, Chronic Obstructive Pulmonary Disease. Principal diagnoses were grouped based on ICD-10 codes into clinically meaningful categories using Clinical Classification Software Refined from the Healthcare Cost and Utilization Project.

**Figure 3:**
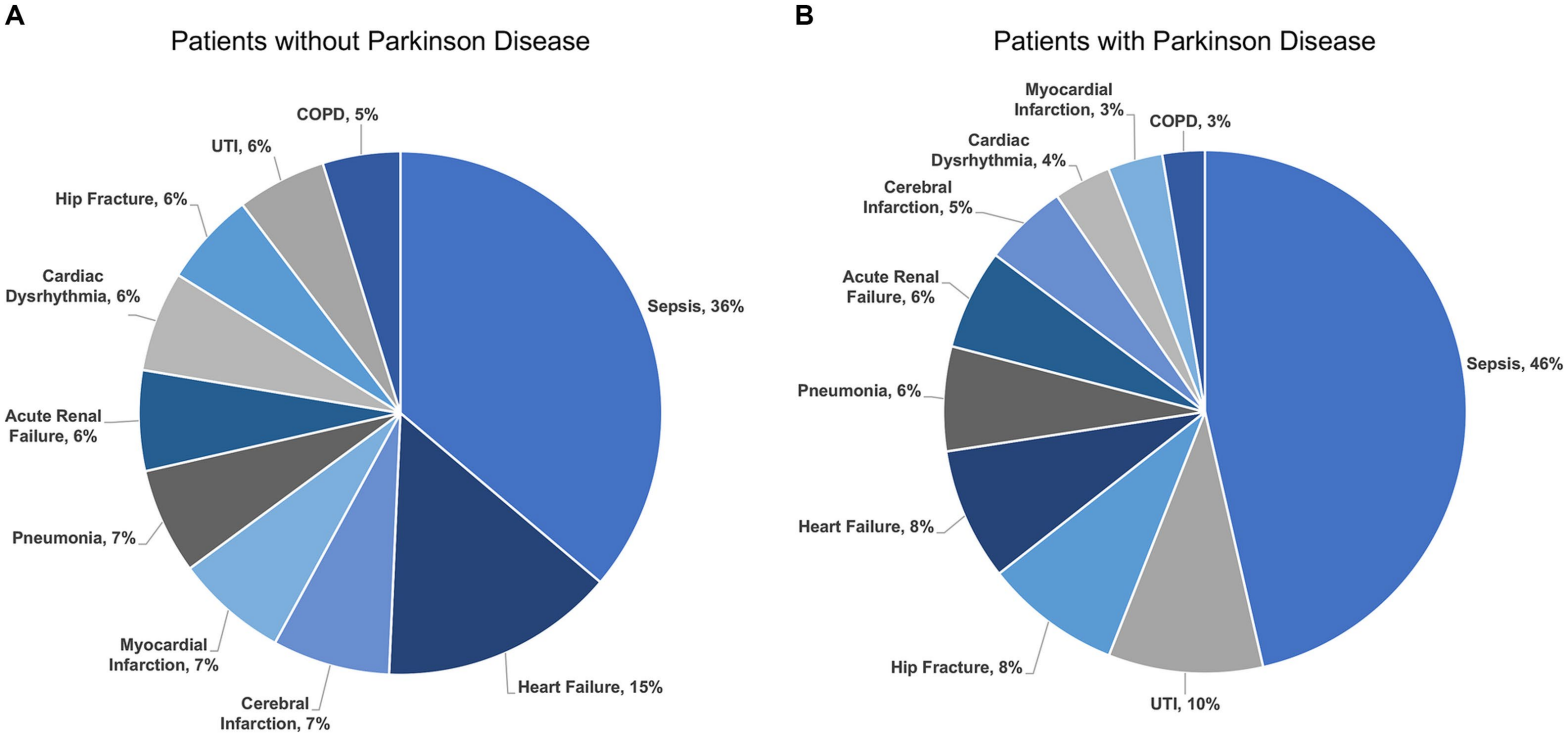
Indications for hospitalization among unmatched older adults **(A)** without Parkinson disease and **(B)** with Parkinson disease by percent. UTI, Urinary Tract Infection; COPD, Chronic Obstructive Pulmonary Disease.

There were 1,232,617 hospitalizations (Figure 1) admitted for any one of the ten most common indications for hospitalization among adults ≥65 years of age including sepsis, heart failure, cerebral infarction, myocardial infarction, pneumonia, renal failure, cardiac dysrhythmia, hip fracture, urinary tract infection, and chronic obstructive pulmonary disease (COPD). The rank order of indications for hospitalization among patients with and without PD were different (*p* < 0.001) (Figure 2). The most common indication for hospitalization among older adults with or without PD was sepsis; 16,468 (46.4%) with PD and 433,113 (36.2%) without PD (Figure 3). The second and third leading causes of hospitalization in all older adults without PD was heart failure (*n* = 173,849, 14.5%) and cerebral infarction (*n* = 86,910, 7.3%), respectively (Figure 2A). In contrast, the second and third leading causes for hospitalization in patients with PD was urinary tract infection (*n* = 3,399, 9.6%) and hip fracture (*n* = 2,989, 8.4%), respectively (Figure 2B). There was no difference in admissions for pneumonia (*p* = 0.55) or acute renal failure (*p* = 0.93) (Figure 3). Patients with PD were less often admitted for heart failure, cerebral infarction, cardiac dysrhythmia, myocardial infarction, and COPD (*p* < 0.001 for all).

### Matching

We identified 35,457 patients with PD among the ten leading indications for hospitalization in older adults matched based on principal diagnosis (one-to-one exact matching), age, gender, race and ethnicity, and Elixhauser comorbidity index (one-to-one coarsened exact matching) to hospitalizations without comorbid PD (Figure 1).

Patient and hospital characteristics for those with and without PD in the matched cohort are in Table 1. Patients with comorbid PD more often had comorbid dementia and depression, and less often had cancer, hypertension, diabetes, peripheral vascular disease, and alcohol or drug abuse. Patients with PD were less often from rural locations (2% vs. 3%, *p* < 0.001), and more often admitted from nursing facilities (14% vs. 8%, *p* < 0.001). Patients with PD were more often from higher income ZIP codes (53% vs. 51% in top 50^th^ percentile, *p* < 0.001) and more often insured by Medicare as the primary payer (92% vs. 90%, *p* < 0.001). Patients with PD more often received care in larger, teaching hospitals. There was no difference observed in the availability of neurologic services among hospitals in which patients with PD and those without PD received care (84% for both, *p* = 0.16).

**Table 1:** Patient and hospital characteristics of the matched cohort for patients with and without Parkinson disease.

Characteristics	No Parkinson disease	Parkinson disease	*p*-value
*N* (%)	35,457 (100)	35,457 (100)	
Age in years, median (IQR)	81 (75–86)	81 (75–86)	0.98
Gender, *n* (%)			
Female	14,810 (42)	14,810 (42)	
Male	20,647 (58)	20,647 (58)	1.00
Race and ethnicity^a^, *n* (%)			
Asian or Pacific Islander	4,284 (12)	4,284 (12)	1.00
Black	1,495 (4)	1,495 (4)	
Hispanic	5,971 (17)	5,971 (17)	
White	21,967 (62)	21,967 (62)	
Other	1,740 (5)	1,740 (5)	
Elixhauser comorbidity index^b^, median (IQR)	5 (4–7)	5 (4–7)	1.00
Comorbidities, *n* (%)			
Dementia	8,057 (23)	16,865 (48)	<0.001
Cancer	3,769 (11)	1,795 (5)	<0.001
Hypertension	29,871 (84)	27,309 (77)	<0.001
Diabetes	15,397 (43)	12,658 (36)	<0.001
Peripheral vascular disease	7,386 (21)	5,376 (15)	<0.001
Alcohol or drug abuse	1,811 (5)	952 (3)	<0.001
Depression	4,711 (13)	6,400 (18)	<0.001
Patient location, *n* (%)			
Urban	34,211 (97)	34,602 (98)	<0.001
Rural	980 (3)	727 (2)	
Point of origin^c^, *n* (%)			
Community	29,704 (84)	27,921 (79)	<0.001
Nursing facility	2,685 (8)	4,940 (14)	
Other healthcare facility	3,035 (9)	2,567 (7)	
Median household income by patient ZIP^d^, *n* (%)			
1st Quartile	8,404 (24)	7,810 (22)	<0.001
2nd Quartile	8,753 (25)	8,523 (24)	
3rd Quartile	9,383 (27)	9,819 (28)	
4th Quartile	8,231 (24)	8,839 (25)	
Primary insurance payer, *n* (%)			
Medicare	32,080 (90)	32,590 (92)	
Medicaid	1,601 (5)	1,324 (4)	
Private	1,210 (3)	1,012 (3)	
Self pay/no charge/other	566 (2)	531 (2)	
Hospital bedsize, *n* (%)			
<100	2,348 (7)	2,470 (7)	0.01
100–199	7,962 (22)	7,817 (22)	
200–299	8,290 (23)	8,259 (23)	
300–399	8,665 (24)	8,350 (24)	
≥400	8,192 (23)	8,561 (24)	
Hospital teaching status, *n* (%)			
Teaching	22,491 (63)	23,129 (65)	<0.001
Non-teaching	12,966 (37)	12,328 (35)	
Neurologic service in hospital, *n* (%)			
No	4,611 (16)	4,702 (16)	0.16
Yes	25,050 (84)	24,748 (84)	

a “Other” race and ethnicity includes individuals not categorized by the database, including those identified as multiple race, not classified, or unknown. Individuals identified as Native American or Alaskan Native are included within this group for confidentiality reasons due to fewer than 10 records within the sample.

b Elixhauser comorbidity index is a measure of comorbidity for use with large administrative datasets with higher numbers representing the presence of greater comorbidity, accounting for up to 31 categories of disease.

c Point of origin is derived from the UB-04 claim form. Other healthcare facilities include clinics, physician offices, ambulatory surgical centers, other acute care hospitals, and non-acute care units within the same hospitals. There were *n* = 33 (0.1%) unknown without PD and *n* = 29 (0.1%) unknown with PD.

d Household income quartiles were assigned based on the median income of the patient’s ZIP Code where the first quartile is the lowest income and fourth quartile is the highest income.

### Potentially preventable complications

**Figure 4:**
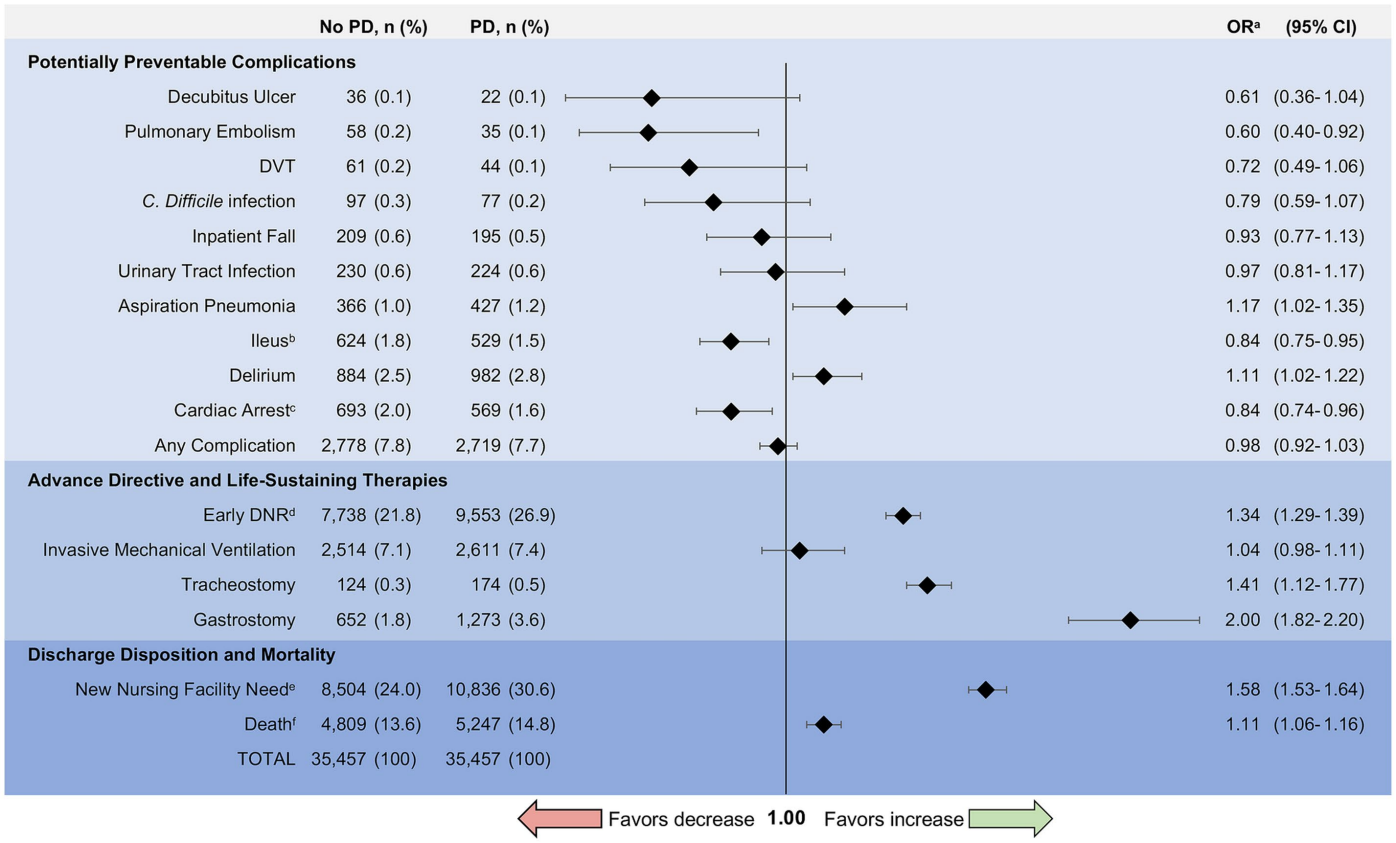
Complications and outcomes for matched patients with and without Parkinson disease. PD, Parkinson disease; OR, Odds Ratio; DVT, Deep Venous Thrombosis; *C. difficile*, *Clostridium difficile*; DNR, Do-Not-Resuscitate. ^a^Odds ratios were calculated using conditional logistic regression to assess the association between comorbid Parkinson disease and complications or outcomes in the matched cohort. ^b^Ileus includes paralytic ileus as well as other reduced motility gastrointestinal complications. ^c^Identifies in-hospital cardiac arrest. Excludes individuals with DNR orders to avoid bias from advance directives. ^d^Early DNR includes patients with DNR orders placed within 24 h of admission to the hospital. ^e^New nursing facility need analyzes discharges to a nursing facility but excludes individuals admitted from a nursing home to avoid bias from point of origin. ^f^Death includes inpatient mortality and discharge to hospice (i.e., total mortality equivalence).

The incidence of complications in both groups was generally low (any complication 7.8% without PD vs. 7.7% with PD, *p* = 0.41) (Figure 4). Hospitalized patients with PD were more likely to experience delirium (Odds Ratio [OR] 1.11, 95% CI 1.02–1.22) and aspiration pneumonia (OR 1.17, 95% CI 1.02–1.35) compared to matched controls. Patients with PD were less likely to develop pulmonary embolism (OR 0.6, 95% CI 0.40–0.92) and ileus or other reduced motility gastrointestinal complications (OR 0.84, 95% CI 0.75–0.95) while in the hospital. In-hospital cardiac arrest (excluding those with DNR orders) was less likely in hospitalized patients with PD compared to those without PD (OR 0.84, 95% CI 0.74–0.96). There was no difference in inpatient falls (OR 0.93, 95% CI 0.77–1.13) or DVT (OR 0.72, 95% CI 0.49–1.06) for hospitalizations with PD compared to those without PD in the matched cohort. Hospitalized patients with PD had similar risk of developing urinary tract infections, *C. difficile* infections, and decubitus ulcers compared to matched controls (Figure 4).

### Advance directive and life-sustaining therapies

PD patients who were hospitalized had greater odds of early DNR orders (OR 1.34, 95% CI 1.29–1.39) (Figure 4). Odds of tracheostomy (OR 1.41, 95% CI 1.12–1.77) and gastrostomy (OR 2.00, 95% CI 1.82–2.2) were increased in hospitalized patients with PD, whereas the odds of invasive mechanical ventilation were similar (OR 1.04, 95% CI 0.98–1.11).

### Discharge disposition and mortality

**Figure 5:**
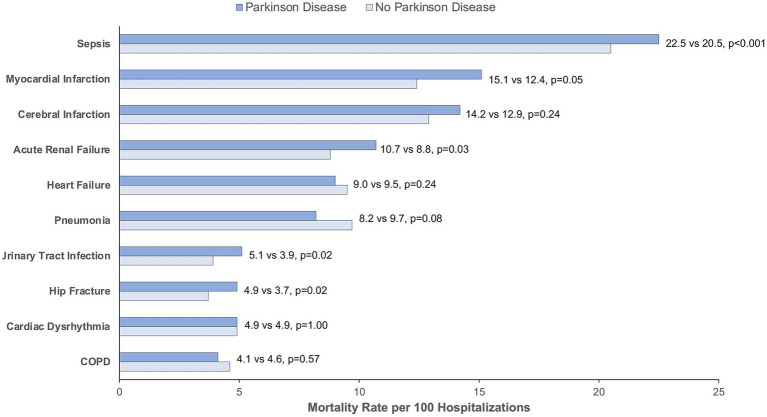
Mortality rate by indication for hospitalization for matched patients with and without Parkinson disease. COPD, Chronic Obstructive Pulmonary Disease. Mortality rate is calculated per 100 hospitalizations for Parkinson disease vs. no Parkinson disease. *p* < 0.05 is considered statistically significant.

There was no difference in length of stay for patients with and without PD (PD: median 4 days, IQR 3–7 days vs. without PD: median 4 days, IQR 2–7 days; *p* = 0.18). Approximately 14.8% of hospitalized patients with PD died compared with 13.6% of hospitalized patients without comorbid PD (OR 1.11, 95% CI 1.06–1.16) (Figure 4). This difference was largely driven by hospice discharge which represented 47% of deaths for patients with PD compared to 37% of deaths in patients without PD (*p* < 0.001). In subgroup analysis, there was greater odds of death for patients with PD compared to those without PD among admissions for sepsis (OR 1.12, 95% CI 1.07–1.19), urinary tract infection (OR 1.32, 95% CI 1.05–1.66), hip fracture (OR 1.35, 95% CI 1.05–1.74), and acute renal failure (OR 1.25, 95% CI 1.02–1.53). Mortality rates by indication for hospitalization can be found in Figure 5. Exclusion of those with early DNR orders did not change the magnitude or direction of the association between comorbid PD and death. Additionally, patients with PD had greater odds of new nursing facility requirement upon discharge (30.6% vs. 24.0%, *p* < 0.001; OR 1.58, 95% CI 1.53–1.64), excluding those who were admitted from nursing facilities.

## Discussion

Several studies to date have found higher rates of hospital admission for patients with PD (Klein et al., 2009; Mueller et al., 2009; Rhalimi et al., 2009); however, prior to this report little was known about inpatient complications and outcomes experienced for patients with PD from large population-based data. In this retrospective observational study of the ten most common indications for hospitalization among older adults in California, we found differences in leading reasons for hospitalization, in-hospital complications, life-sustaining therapy utilization, and outcomes for patients with PD.

First, we found that older adults with PD similarly experienced admissions for sepsis as the leading cause of hospitalization in comparison to older adults without PD; however, sepsis accounted for a greater share of admissions for PD patients. While hip fracture and urinary tract infections represented the 8th and 9th leading causes of hospitalization among older adults without PD, these diagnoses were the 3rd and 2nd leading causes for hospitalizations among patients with PD, respectively. Prior studies used varied data sources and methods to identify indications for hospitalization among patients with PD (Woodford and Walker, 2005; Temlett and Thompson, 2006; Klein et al., 2009; Vossius et al., 2010; Gil-Prieto et al., 2016; Mahajan et al., 2016; Okunoye et al., 2020), however, few had comparisons to patients without PD (Vossius et al., 2010). Based on our results, sepsis, urinary tract infections, and hip fractures related to falls may be important targets for future study on PD-associated hospitalizations.

Additionally, while our cohort of hospitalized older adults with PD and those without PD were matched on Elixhauser comorbidity index (a summary metric which quantifies comorbidity), the profile of comorbidities differed between the two groups. Comorbidities such as cancer, hypertension, diabetes, peripheral vascular disease, and alcohol or drug abuse were more likely to be observed in hospitalized patients without PD, whereas dementia and depression were more common among hospitalized patients with PD. Furthermore, comorbidities among hospitalized patients with and without PD may be different than observations of comorbid disease in the community. For example, PD is associated with an increased prevalence of diabetes in the community (Cereda et al., 2011), differing from our study of hospitalized patients which demonstrates those without PD were more likely to have diabetes.

Second, patients with PD had differences in risk of complications during hospitalization. We found an increased odds of developing aspiration pneumonia as a complication of hospitalization compared to matched controls. Between 32% and 70% of patients with PD have dysphagia, or swallowing difficulties (Miller et al., 2009). Few studies have examined aspiration as a complication of inpatient stays for PD (Temlett and Thompson, 2006; Miller et al., 2009), however, the high risk of dysphagia (Miller et al., 2009; Suttrup and Warnecke, 2016), potential for greater immobility in the hospital (Aminoff et al., 2011; Oguh and Videnovic, 2012), and increased risk of cognitive changes (acute or chronic) (Williams-Gray et al., 2007; Dham et al., 2023) may subject patients with PD to higher risk of aspiration while hospitalized. Based on these results, patients with PD may benefit from routine and rigorous inpatient monitoring of swallow function during their hospital stay and early use of short-term alternatives to feeding when indicated (e.g., nasogastric tubes); similar to the management of stroke patients (Dennis et al., 2005; Dziewas et al., 2021).

Our study also identified an increased risk of delirium for hospitalizations with comorbid PD. Potential precipitants of delirium in hospitalized patients with PD include infections, recent surgery, urinary retention, constipation, pain, metabolic abnormalities, and mismanagement of PD-related medications or administration of contraindicated medications (Golden et al., 1989; Price et al., 2015; Marcantonio, 2017; Dham et al., 2023). Patients with PD are susceptible to in-hospital medication errors such as missed or poorly timed doses of PD-related medications (e.g., Carbidopa/Levodopa) or abrupt discontinuation of PD medications either unintentionally or due to lack of *Per Os* access (Magdalinou et al., 2007; Buetow et al., 2010; Derry et al., 2010; Hou et al., 2012). Patients with PD may also receive contraindicated medications during their hospitalization, such as centrally-acting antiemetics, neuroleptics, or others (Keyser and Rodnitzky, 1991; Rhalimi et al., 2009). Prevention of these in-hospital medication errors are identified as recommended care standards for hospitalized PD patients within the Parkinson’s Foundation Hospital Care Recommendations (2023). Although the clinical granularity of our study is limited in the ability to fully elucidate contributing factors to delirium, our findings are consistent with that of prior studies examining risk of in-hospital delirium for patients with PD (Golden et al., 1989; Dham et al., 2023).

While reduced mobility and motor disturbances is likely a greater issue for patients with PD during their hospitalization (Woodford and Walker, 2005; Klein et al., 2009), we unexpectedly found individuals with PD were less likely to receive a diagnosis of pulmonary embolism and ileus as a complication of their inpatient stay, and they experienced inpatient falls at similar rates compared to matched controls. There is potential for this association to be related to detection or reporting bias. It is possible that patients with PD could be subject to therapeutic nihilism, similar to that observed in other chronic neurodegenerative conditions (Sedney et al., 2019; Maksymowicz et al., 2022). A diagnosis of pulmonary embolism and ileus may require further diagnostics beyond a bedside clinical evaluation (Weledji, 2020; Cafferkey et al., 2022), which is unlikely to be ordered for a patient with an anticipated poor outcome or one who awaits hospice discharge. Inpatient falls in which no injury was sustained may be underappreciated, and may be viewed as a common or expected occurrence rather than an adverse outcome (Oliver, 2004; Haines et al., 2008). Unfortunately, our data rely on recorded diagnosis codes for hospitalizations, and we are unable to account for imaging or other testing during an inpatient stay using the SID.

Third, we found greater odds of early DNR orders and decreased odds of in-hospital cardiac arrest, even when those with DNR orders were excluded. Consistent with prior studies (Mahajan et al., 2017), patients with PD were more often admitted with DNR orders underscoring the typical feelings of patients and their surrogates toward resuscitative efforts on admission in the setting of chronic neurodegenerative illness (Gaster et al., 2017). When patients with DNR orders were excluded, there were still decreased odds of in-hospital cardiac arrest with resuscitative efforts in hospitalized patients with comorbid PD compared to those without PD. While this finding could potentially be a marker for lower overall disease severity despite matching, this more likely represents patient, surrogate, and provider attitudes toward resuscitation in patients with neurodegenerative illness. Further study is needed to better understand the characteristics and decision-making processes for critically ill patients with PD forgoing cardiopulmonary resuscitative efforts.

Fourth, we found that patients with PD receive gastrostomy and tracheostomy more often compared to matched controls. Studies in older adults with dementia have shown no improvement in the risks of aspiration, nutritional deficiency, or death with gastrostomy placement (Finucane et al., 1999; McCann, 1999). Despite the prior evidence, patients with PD appear to receive gastrostomy more often in our study, presumably for sustainable artificial nutrition and prevention of aspiration. There is no evidence to date demonstrating outcomes (e.g., aspiration occurrence, survival) specific to patients with PD undergoing gastrostomy placement. However, surgical feeding tube placement is a preference-sensitive decision for patients and surrogates (O'Brien et al., 1997), which is subject to wide geographic and institutional practice variability (George et al., 2014; Hwang et al., 2018). Potential logistical benefits of surgical feeding tubes may include consistent medication administration and eligibility for nursing facility placement which may reduce length of stay (in contrast to more temporary nasogastric feeding tubes) (George et al., 2017).

Little is known about the use of tracheostomy for patients with PD, although there has been a proposed benefit for patients with Parkinson-plus syndromes and airway dysfunction (Sinclair et al., 2013). Our study appears to be the first to date that has found an increased odds of receiving tracheostomy among hospitalized patients with PD. Given the elevated risk of aspiration and potential for accompanying respiratory failure, it may be expected that patients with PD receive tracheostomy with greater frequency. Furthermore, issues with upper airway musculature in PD may play a role in mediating airway dysfunction, further complicating ventilator liberation for patients with PD (Vincken et al., 1984). However, like gastrostomy, indications for tracheostomy are typically accompanied by severe functionally disabling illness as well as considerable healthcare costs (Engoren et al., 2004; Seder, 2019; Wahlster et al., 2021), and could be compounded by a progressive neurodegenerative disorder for patients with PD. Therefore, further study is needed to understand recent clinical practices, patient and surrogate preferences, and the associated value with use of life-sustaining therapies in the setting of hospitalized patients with PD.

Fifth, patients with PD experience death (as measured by inpatient mortality or hospice discharge – i.e., total mortality equivalence) (Asch et al., 2021) more often than matched controls. This association held even with the exclusion of individuals with DNR orders. However, hospice appeared to be an important determinant with nearly half of those counted as PD deaths undergoing discharge to hospice. In this regard, deaths may be overestimated since there are likely some patients who experience “live discharge” from hospice in time (Dolin et al., 2017), but this methodology is needed in light of increasing hospice utilization by hospitals to improve mortality statistics (Marks, 2015). When examined within the subgroups by indication for hospitalization, those admitted for sepsis, acute renal failure, hip fracture, and urinary tract infection demonstrated greater mortality compared to matched controls. There was a non-significant trend towards greater mortality for those admitted with myocardial infarction. These indications for hospitalization may represent reasonable targets for interventions that may aim to improve outcomes for hospitalized individuals with PD.

Previous studies have identified inpatient mortality rates between 4 and 10% for hospitalized patients with PD (Pepper and Goldstein, 1999; Gil-Prieto et al., 2016; Mahajan et al., 2016; Rumalla et al., 2017), compared to our study which identified an inpatient mortality rate of approximately 15% among hospitalized patients with PD. Accounting for hospice discharge in the mortality rate may account for the greater mortality observed in our study compared to prior findings (Sleeman et al., 2013).

Finally, patients with PD demonstrated greater odds of new nursing facility requirement upon discharge. Discharge to a nursing facility is often needed for patients that require a higher level of post-acute care, and greater assistance with activities of daily living, that may not be feasible at home (Quine and Morrell, 2007). This finding indicates a greater loss of independence following a hospitalization among patients with PD compared to matched controls. This highlights a need to better understand loss of independence for hospitalized patients with PD, and to develop interventions that will improve safe living while preserving autonomy and patient satisfaction (Leff et al., 2005; Csipke et al., 2021).

### Limitations

There were several limitations to our study. Our study relies on ICD-10 codes for the identification of diagnoses, complications, and procedures within administrative hospital data. These codes are subject to misidentification and under or over-reporting based on the inherent inaccuracies of administrative data. However, we used ICD-10 codes akin to that previously used in studies of administrative datasets for Parkinson disease (Willis et al., 2012; Dham et al., 2023). We used procedure codes associated with highly billable services (e.g., tracheostomy, gastrostomy) which are unlikely to be missed in administrative records (George et al., 2014, 2017; Albert et al., 2023), and the 25 available procedural fields for hospitalizations within the matched cohort were rarely full (*n* = 32 of 70,914 patients with all 25 fields documented). Additionally, we used a coding methodology and adjunct POA indicators that are industry standard in the identification of potentially preventable complications (PPCs) (Hughes et al., 2006; Lagoe and Bick, 2013; 3M™ Health Care Academy, 2023; Health Data NY, 2023). There are a few likely incentives for hospitals to under-report complications (e.g., code actual complications as present on admission): (1) to reduce complication rates, and (2) to increase severity of illness on admission. The extent to which under-reporting occurs is unknown. While we cannot ensure complete accuracy, we ameliorated potential errors by excluding hospitals with poor reporting (<5% of mandatory reportable diagnoses) of POA indicators, selecting only those hospitals with high compliance in POA-reportable diagnoses. Unfortunately, we are unable to determine the exact timing of inpatient complications as they relate to the day of admission, only that they were not present on admission. We cannot exclude the possibility that some PPCs are related to the reason for hospitalization such as a complication that develops in sequence as a result of an admitting diagnosis (e.g., cardiac arrest that occurs days after an admission for septic shock), and there is no way to know the extent to which the admitting diagnosis contributed to the development of a complication using these data. Furthermore, our study lacks granular clinical data such as exam findings, lab values, specific medication administration and dosing, deep brain stimulation status, or disease severity metrics commonly used for PD. Our study was limited to inpatient hospitalization data used in California, and therefore, our results may not be generalizable to larger populations of patients with PD. For our study, we chose to focus on older adults given the large proportion of PD patients within this cohort and the intrinsic differences in common reasons for hospitalization for younger versus older adults. Therefore, our study may not be applicable to the young adult PD population. Finally, our study is cross sectional at the time point of each hospitalization; we are unable to follow patients longitudinally, and therefore, outcomes following discharge are unknown.

Prior studies evaluating PD hospitalization outcomes are often limited by the degree of power and scope of the patient populations investigated. Despite the our study’s limitations, this is (to our knowledge) the first study of inpatient complications and outcomes of this magnitude with analysis of over 35,000 hospitalized patients with PD admitted to institutions ranging from large academic centers, non-federal government-owned facilities, and urban or rural community hospitals, encompassing all insurance payers, and including diverse groups who are frequently under-represented in the current literature.

## Conclusion

Patients with PD are at greater risk of developing aspiration pneumonia and delirium as a complication of their hospitalization. Patients with PD more often have early DNR orders and they experience in-hospital cardiac arrest less often. However, hospitalized patients with comorbid PD demonstrated greater utilization of life-sustaining therapies. Furthermore, patients with PD experience worse outcomes of their hospitalization including new nursing facility requirements indicating a loss of independence following discharge, and greater odds of death resulting from hospitalization. Further study is needed to identify interventions that will improve care, optimize patient-centered decision-making, and ultimately, generate better outcomes for patients hospitalized with PD.

## Data availability statement

The data analyzed in this study is subject to the following licenses/restrictions: available for purchase under HCUP licensing agreement. Requests to access these datasets should be directed to HCUP-RequestData@ahrq.gov.

## Ethics statement

The studies involving humans were approved by University of Rochester Research Subjects Review Board. The studies were conducted in accordance with the local legislation and institutional requirements. Written informed consent for participation was not required from the participants or the participants’ legal guardians/next of kin in accordance with the national legislation and institutional requirements.

## Author contributions

BG: Conceptualization, Data curation, Formal analysis, Investigation, Methodology, Resources, Software, Visualization, Writing – original draft, Writing – review & editing. WB: Formal analysis, Investigation, Writing – review & editing. AS: Formal analysis, Investigation, Writing – original draft. IR: Conceptualization, Formal analysis, Funding acquisition, Investigation, Supervision, Writing – review & editing.
