# Supplementary material for: Complications and outcomes of hospitalizations for patients with and without Parkinson disease
Source: Front Aging Neurosci. 2023 Dec 15;15:1276731. doi: 10.3389/fnagi.2023.1276731 (PMC10757345; doi:10.3389/fnagi.2023.1276731)
Supplement: Supplementary file 1 [file Data_Sheet_1.PDF]

**Table S1.** Identification of Potentially Preventable Complications

| Potentially Preventable Complications[a] | ICD-10 Code | Description | Exclusions[b] |
|---|---|---|---|
| Delirium | F05 | Delirium due to known physiological condition | |
| | R41.0 | Disorientation, unspecified | |
| | R41.82 | Altered Mental Status, unspecified | |
| | G92.8 | Other toxic encephalopathy | |
| | G92.9 | Unspecified toxic encephalopathy | |
| | G93.40 | Encephalopathy, unspecified | |
| | G93.41 | Metabolic encephalopathy | |
| | G93.49 | Other encephalopathy | |
| Aspiration Pneumonia | J69.0 | Pneumonitis due to inhalation of food and vomit | Principal Diagnosis of Pneumonia CCSR code RSP002 |
| | J69.1 | Pneumonitis due to inhalation of oils and essences | |
| | J69.8 | Pneumonitis due to inhalation of other solids and liquids | |
| Cardiac Arrest | I46.2 | Cardiac arrest due to underlying cardiac condition | |
| | I46.8 | Cardiac arrest due to other underlying condition | |
| | I46.9 | Cardiac arrest, cause unspecified | |
| | I49.01 | Ventricular fibrillation | |
| | I49.02 | Ventricular flutter | |
| | 5A12012 | Performance of Cardiac Output, Single, Manual | |
| | 5A1221J | Performance of Cardiac Output, Continuous, Automated | |
| *C. Difficile* Infection | A04.71 | Enterocolitis due to Clostridium difficile, recurrent | |
| | A04.72 | Enterocolitis due to Clostridium difficile, not specified as recurrent | |
| Deep Venous Thrombosis | I80.10 | Phlebitis and thrombophlebitis of unspecified femoral vein | |
| | I80.11 | Phlebitis and thrombophlebitis of right femoral vein | |
| | I80.12 | Phlebitis and thrombophlebitis of left femoral vein | |
| | I80.13 | Phlebitis and thrombophlebitis of femoral vein, bilateral | |
| | I80.201 | Phlebitis and thrombophlebitis of unspecified deep vessels of right lower extremity | |
| | I80.202 | Phlebitis and thrombophlebitis of unspecified deep vessels of left lower extremity | |
| | I80.203 | Phlebitis and thrombophlebitis of unspecified deep vessels of lower extremities, bilateral | |
| | I80.209 | Phlebitis and thrombophlebitis of unspecified deep vessels of unspecified lower extremity | |
| | I80.211 | Phlebitis and thrombophlebitis of right iliac vein | |
| | I80.212 | Phlebitis and thrombophlebitis of left iliac vein | |
| | I80.213 | Phlebitis and thrombophlebitis of iliac vein, bilateral | |
| | I80.219 | Phlebitis and thrombophlebitis of unspecified iliac vein | |
| | I80.221 | Phlebitis and thrombophlebitis of right popliteal vein | |
| | I80.222 | Phlebitis and thrombophlebitis of left popliteal vein | |
| | I80.223 | Phlebitis and thrombophlebitis of popliteal vein, bilateral | |
| | I80.229 | Phlebitis and thrombophlebitis of unspecified popliteal vein | |
| | I80.291 | Phlebitis and thrombophlebitis of other deep vessels of right lower extremity | |
| | I80.292 | Phlebitis and thrombophlebitis of other deep vessels of left lower extremity | |
| | I80.293 | Phlebitis and thrombophlebitis of other deep vessels of lower extremity, bilateral | |
| | I80.299 | Phlebitis and thrombophlebitis of other deep vessels of unspecified lower extremity | |
| | I80.3 | Phlebitis and thrombophlebitis of lower extremities, unspecified | |
| | I80.9 | Phlebitis and thrombophlebitis of unspecified site | |
| | I82.401 | Acute embolism and thrombosis of unspecified deep veins of right lower extremity | |

| | | | |
|---|---|---|---|
| | I82.402 | Acute embolism and thrombosis of unspecified deep veins of left lower extremity | |
| | I82.403 | Acute embolism and thrombosis of unspecified deep veins of lower extremity, bilateral | |
| | I82.409 | Acute embolism and thrombosis of unspecified deep veins of unspecified lower extremity | |
| | I82.411 | Acute embolism and thrombosis of right femoral vein | |
| | I82.412 | Acute embolism and thrombosis of left femoral vein | |
| | I82.413 | Acute embolism and thrombosis of femoral vein, bilateral | |
| | I82.419 | Acute embolism and thrombosis of unspecified femoral vein | |
| | I82.421 | Acute embolism and thrombosis of right iliac vein | |
| | I82.422 | Acute embolism and thrombosis of left iliac vein | |
| | I82.423 | Acute embolism and thrombosis of iliac vein, bilateral | |
| | I82.429 | Acute embolism and thrombosis of unspecified iliac vein | |
| | I82.431 | Acute embolism and thrombosis of right popliteal vein | |
| | I82.432 | Acute embolism and thrombosis of left popliteal vein | |
| | I82.433 | Acute embolism and thrombosis of popliteal vein, bilateral | |
| | I82.439 | Acute embolism and thrombosis of unspecified popliteal vein | |
| | I82.491 | Acute embolism and thrombosis of other specified deep vein of right lower extremity | |
| | I82.492 | Acute embolism and thrombosis of other specified deep vein of left lower extremity | |
| | I82.493 | Acute embolism and thrombosis of other specified deep vein of lower extremity, bilateral | |
| | I82.499 | Acute embolism and thrombosis of other specified deep vein of unspecified lower extremity | |
| | I82.4Y1 | Acute embolism and thrombosis of unspecified deep veins of right proximal lower extremity | |
| | I82.4Y2 | Acute embolism and thrombosis of unspecified deep veins of left proximal lower extremity | |
| | I82.4Y3 | Acute embolism and thrombosis of unspecified deep veins of proximal lower extremity, bilateral | |
| | I824Y9 | Acute embolism and thrombosis of unspecified deep veins of unspecified proximal lower extremity | |
| | T81.72xA | Complication of vein following a procedure, not elsewhere classified, initial encounter | |
| Falls | W[01][0123456789][c] | Slipping, tripping, stumbling and falls | Absence of comorbid diagnoses ICD-10-CM code Y92.23 "Hospital as place of occurrence for external cause of injury" |
| Ileus | K91.3 | Postprocedural intestinal obstruction | |
| | K56 | Paralytic ileus and intestinal obstruction without hernia | |
| | K59.81 | Ogilvie syndrome | |
| | K59.00 | Constipation unspecified | |
| | K59.01 | Slow transit constipation | |
| | K59.02 | Outlet dysfunction constipation | |
| | K59.03 | Drug induced constipation | |
| | K59.09 | Other constipation | |

| | | | |
|---|---|---|---|
| Pulmonary Embolism | I26.01 | Septic pulmonary embolism with acute cor pulmonale | |
| | I26.02 | Saddle embolus of pulmonary artery with acute cor pulmonale | |
| | I26.09 | Other pulmonary embolism with acute cor pulmonale | |
| | I26.90 | Septic pulmonary embolism without acute cor pulmonale | |
| | I26.92 | Saddle embolus of pulmonary artery without acute cor pulmonale | |
| | I26.93 | Single subsegmental pulmonary embolism without acute cor pulmonale | |
| | I26.94 | Multiple subsegmental pulmonary emboli without acute cor pulmonale | |
| | I26.99 | Other pulmonary embolism without acute cor pulmonale | |
| Urinary Tract Infection | N30.00 | Acute cystitis without hematuria | Principal Diagnosis of UTI CCSR code GEN004 |
| | N30.01 | Acute cystitis with hematuria | |
| | N30.80 | Other cystitis without hematuria | |
| | N30.90 | Cystitis, unspecified without hematuria | |
| | N30.91 | Cystitis, unspecified with hematuria | |
| | N39.0 | Urinary tract infection, site not specified | |
| | N41.3 | Prostatocystitis | |
| Decubitus Ulcer | L89.000 | Pressure ulcer of unspecified elbow, unstageable | Length of Stay ≤4 days |
| | L89.003 | Pressure ulcer of unspecified elbow, stage 3 | |
| | L89.004 | Pressure ulcer of unspecified elbow, stage 4 | |
| | L89.006 | Pressure-induced deep tissue damage of unspecified elbow | |
| | L89.010 | Pressure ulcer of right elbow, unstageable | |
| | L89.013 | Pressure ulcer of right elbow, stage 3 | |
| | L89.014 | Pressure ulcer of right elbow, stage 4 | |
| | L89.016 | Pressure-induced deep tissue damage of right elbow | |
| | L89.020 | Pressure ulcer of left elbow, unstageable | |
| | L89.023 | Pressure ulcer of left elbow, stage 3 | |
| | L89.024 | Pressure ulcer of left elbow, stage 4 | |
| | L89.026 | Pressure-induced deep tissue damage of left elbow | |
| | L89.100 | Pressure ulcer of unspecified part of back, unstageable | |
| | L89.103 | Pressure ulcer of unspecified part of back, stage 3 | |
| | L89.104 | Pressure ulcer of unspecified part of back, stage 4 | |
| | L89.106 | Pressure-induced deep tissue damage of unspecified part of back | |
| | L89.110 | Pressure ulcer of right upper back, unstageable | |
| | L89.113 | Pressure ulcer of right upper back, stage 3 | |
| | L89.114 | Pressure ulcer of right upper back, stage 4 | |
| | L89.116 | Pressure-induced deep tissue damage of right upper back | |
| | L89.120 | Pressure ulcer of left upper back, unstageable | |
| | L89.123 | Pressure ulcer of left upper back, stage 3 | |
| | L89.124 | Pressure ulcer of left upper back, stage 4 | |
| | L89.126 | Pressure-induced deep tissue damage of left upper back | |
| | L89.130 | Pressure ulcer of right lower back, unstageable | |
| | L89.133 | Pressure ulcer of right lower back, stage 3 | |
| | L89.134 | Pressure ulcer of right lower back, stage 4 | |
| | L89.136 | Pressure-induced deep tissue damage of right lower back | |
| | L89.140 | Pressure ulcer of left lower back, unstageable | |
| | L89.143 | Pressure ulcer of left lower back, stage 3 | |
| | L89.144 | Pressure ulcer of left lower back, stage 4 | |
| | L89.146 | Pressure-induced deep tissue damage of left lower back | |
| | L89.150 | Pressure ulcer of sacral region, unstageable | |
| | L89.153 | Pressure ulcer of sacral region, stage 3 | |
| | L89.154 | Pressure ulcer of sacral region, stage 4 | |
| | L89.156 | Pressure-induced deep tissue damage of sacral region | |
| | L89.200 | Pressure ulcer of unspecified hip, unstageable | |
| | L89.203 | Pressure ulcer of unspecified hip, stage 3 | |
| | L89.204 | Pressure ulcer of unspecified hip, stage 4 | |
| | L89.206 | Pressure-induced deep tissue damage of unspecified hip | |

| | | | |
|---|---|---|---|
| | L89.210 | Pressure ulcer of right hip, unstageable | |
| | L89.213 | Pressure ulcer of right hip, stage 3 | |
| | L89.214 | Pressure ulcer of right hip, stage 4 | |
| | L89.216 | Pressure-induced deep tissue damage of right hip | |
| | L89.220 | Pressure ulcer of left hip, unstageable | |
| | L89.223 | Pressure ulcer of left hip, stage 3 | |
| | L89.224 | Pressure ulcer of left hip, stage 4 | |
| | L89.226 | Pressure-induced deep tissue damage of left hip | |
| | L89.300 | Pressure ulcer of unspecified buttock, unstageable | |
| | L89.303 | Pressure ulcer of unspecified buttock, stage 3 | |
| | L89.304 | Pressure ulcer of unspecified buttock, stage 4 | |
| | L89.306 | Pressure-induced deep tissue damage of unspecified buttock | |
| | L89.310 | Pressure ulcer of right buttock, unstageable | |
| | L89.313 | Pressure ulcer of right buttock, stage 3 | |
| | L89.314 | Pressure ulcer of right buttock, stage 4 | |
| | L89.316 | Pressure-induced deep tissue damage of right buttock | |
| | L89.320 | Pressure ulcer of left buttock, unstageable | |
| | L89.323 | Pressure ulcer of left buttock, stage 3 | |
| | L89.324 | Pressure ulcer of left buttock, stage 4 | |
| | L89.326 | Pressure-induced deep tissue damage of left buttock | |
| | L89.43 | Pressure ulcer of contiguous site of back, buttock and hip, stage 3 | |
| | L89.44 | Pressure ulcer of contiguous site of back, buttock and hip, stage 4 | |
| | L89.45 | Pressure ulcer of contiguous site of back, buttock and hip, unstageable | |
| | L89.46 | Pressure-induced deep tissue damage of contiguous site of back, buttock and hip | |
| | L89.500 | Pressure ulcer of unspecified ankle, unstageable | |
| | L89.503 | Pressure ulcer of unspecified ankle, stage 3 | |
| | L89.504 | Pressure ulcer of unspecified ankle, stage 4 | |
| | L89.506 | Pressure-induced deep tissue damage of unspecified ankle | |
| | L89.510 | Pressure ulcer of right ankle, unstageable | |
| | L89.513 | Pressure ulcer of right ankle, stage 3 | |
| | L89.514 | Pressure ulcer of right ankle, stage 4 | |
| | L89.516 | Pressure-induced deep tissue damage of right ankle | |
| | L89.520 | Pressure ulcer of left ankle, unstageable | |
| | L89.523 | Pressure ulcer of left ankle, stage 3 | |
| | L89.524 | Pressure ulcer of left ankle, stage 4 | |
| | L89.526 | Pressure-induced deep tissue damage of left ankle | |
| | L89.600 | Pressure ulcer of unspecified heel, unstageable | |
| | L89.603 | Pressure ulcer of unspecified heel, stage 3 | |
| | L89.604 | Pressure ulcer of unspecified heel, stage 4 | |
| | L89.606 | Pressure-induced deep tissue damage of unspecified heel | |
| | L89.610 | Pressure ulcer of right heel, unstageable | |
| | L89.613 | Pressure ulcer of right heel, stage 3 | |
| | L89.614 | Pressure ulcer of right heel, stage 4 | |
| | L89.616 | Pressure-induced deep tissue damage of right heel | |
| | L89.620 | Pressure ulcer of left heel, unstageable | |
| | L89.623 | Pressure ulcer of left heel, stage 3 | |
| | L89.624 | Pressure ulcer of left heel, stage 4 | |
| | L89.626 | Pressure-induced deep tissue damage of left heel | |
| | L89.810 | Pressure ulcer of head, unstageable | |
| | L89.813 | Pressure ulcer of head, stage 3 | |
| | L89.814 | Pressure ulcer of head, stage 4 | |
| | L89.816 | Pressure-induced deep tissue damage of head | |
| | L89.890 | Pressure ulcer of other site, unstageable | |
| | L89.893 | Pressure ulcer of other site, stage 3 | |
| | L89.894 | Pressure ulcer of other site, stage 4 | |

| | | | |
|---|---|---|---|
| | L89.896 | Pressure-induced deep tissue damage of other site | |
| | L89.93 | Pressure ulcer of unspecified site, stage 3 | |
| | L89.94 | Pressure ulcer of unspecified site, stage 4 | |
| | L89.95 | Pressure ulcer of unspecified site, unstageable | |
| | L89.96 | Pressure-induced deep tissue damage of unspecified site | |

Abbreviations: ICD-10 = International Classification of Diseases, 10$^{th}$ Revision, *C. difficile* = *Clostridium difficile*, CCSR = Clinical Classification Software Refined, UTI = Urinary Tract Infection

a) Complications were only considered present if the record had a corresponding “N” value for the “Present on Admission” indicator.
b) The complication was considered not present for records with an indication for exclusion of the complication.
c) Denotes regular expression of ICD-10 code.

**Table S2.** Identification of Life Sustaining Therapies

| Life-Sustaining Therapies | ICD-10 Code | Description |
|---|---|---|
| Invasive Mechanical Ventilation | 5A1935Z | Respiratory Ventilation, Less than 24 Consecutive Hours |
| | 5A1945Z | Respiratory Ventilation, 24-96 Consecutive Hours |
| | 5A1955Z | Respiratory Ventilation, Greater than 96 Consecutive Hours |
| | 0BH17EZ | Insertion of Endotracheal Airway into Trachea, Via Natural or Artificial Opening |
| | 0BH18EZ | Insertion of Endotracheal Airway into Trachea, Via Natural or Artificial Opening Endoscopic |
| Tracheostomy | 0B110F4 | Bypass Trachea to Cutaneous with Tracheostomy Device, Open Approach |
| | 0B113F4 | Bypass Trachea to Cutaneous with Tracheostomy Device, Percutaneous Approach |
| | 0B114F4 | Bypass Trachea to Cutaneous with Tracheostomy Device, Percutaneous Endoscopic Approach |
| | 0BH13EZ | Insertion of Endotracheal Airway into Trachea, Percutaneous Approach |
| Gastrostomy | 0D16074 | Bypass Stomach to Cutaneous with Autologous Tissue Substitute, Open Approach |
| | 0D160J4 | Bypass Stomach to Cutaneous with Synthetic Substitute, Open Approach |
| | 0D160K4 | Bypass Stomach to Cutaneous with Nonautologous Tissue Substitute, Open Approach |
| | 0D160Z4 | Bypass Stomach to Cutaneous, Open Approach |
| | 0D163J4 | Bypass Stomach to Cutaneous with Synthetic Substitute, Percutaneous Approach |
| | 0D16474 | Bypass Stomach to Cutaneous with Autologous Tissue Substitute, Percutaneous Endoscopic Approach |
| | 0D164K4 | Bypass Stomach to Cutaneous with Nonautologous Tissue Substitute, Percutaneous Endoscopic Approach |
| | 0D16874 | Bypass Stomach to Cutaneous with Autologous Tissue Substitute, Via Natural or Artificial Opening Endoscopic |
| | 0D168J4 | Bypass Stomach to Cutaneous with Synthetic Substitute, Via Natural or Artificial Opening Endoscopic |
| | 0D168K4 | Bypass Stomach to Cutaneous with Nonautologous Tissue Substitute, Via Natural or Artificial Opening Endoscopic |
| | 0D168Z4 | Bypass Stomach to Cutaneous, Via Natural or Artificial Opening Endoscopic |
| | 0DH60UZ | Insertion of Feeding Device into Stomach, Open Approach |
| | 0DH63UZ | Insertion of Feeding Device into Stomach, Percutaneous Approach |
| | 0DH64UZ | Insertion of Feeding Device into Stomach, Percutaneous Endoscopic Approach |
| | 0DH67UZ | Insertion of Feeding Device into Stomach, Via Natural or Artificial Opening |
| | 0DH68UZ | Insertion of Feeding Device into Stomach, Via Natural or Artificial Opening Endoscopic |
| | 0DHA[03478]UZ[a] | Insertion of Feeding Device into Jejunum |

Abbreviations: ICD-10 = International Classification of Diseases, 10th Revision

a) Denotes regular expression of ICD-10 code.
